# A Novel Retinal Oscillation Mechanism in an Autosomal Dominant Photoreceptor Degeneration Mouse Model

**DOI:** 10.3389/fncel.2015.00513

**Published:** 2016-01-12

**Authors:** Hung-Ya Tu, Yu-Jiun Chen, Adam R. McQuiston, Chuan-Chin Chiao, Ching-Kang Chen

**Affiliations:** ^1^Department of Ophthalmology, Baylor College of MedicineHouston, TX, USA; ^2^Institute of Molecular Medicine, National Tsing Hua UniversityHsinchu, Taiwan; ^3^Department of Life Science, National Tsing Hua UniversityHsinchu, Taiwan; ^4^Department of Anatomy and Neurobiology, Virginia Commonwealth UniversityRichmond, VA, USA; ^5^Institute of Systems Neuroscience, National Tsing Hua UniversityHsinchu, Taiwan; ^6^Department of Biochemistry and Molecular Biology, Baylor College of MedicineHouston, TX, USA; ^7^Department of Neuroscience, Baylor College of MedicineHouston, TX, USA

**Keywords:** retina, starburst amacrine cell, AII amacrine cells, oscillation mechanism, photoreceptor degeneration

## Abstract

It has been shown in *rd1* and *rd10* models of photoreceptor degeneration (PD) that inner retinal neurons display spontaneous and rhythmic activities. Furthermore, the rhythmic activity has been shown to require the gap junction protein connexin 36, which is likely located in AII amacrine cells (AII-ACs). In the present study, an autosomal dominant PD model called rhoΔCTA, whose rods overexpress a C-terminally truncated mutant rhodopsin and degenerate with a rate similar to that of *rd1*, was used to investigate the generality and mechanisms of heightened inner retinal activity following PD. To fluorescently identify cholinergic starburst amacrine cells (SACs), the rhoΔCTA mouse was introduced into a combined ChAT-IRES-Cre and Ai9 background. In this mouse, we observed excitatory postsynaptic current (EPSC) oscillation and non-rhythmic inhibitory postsynaptic current (IPSC) in both ON- and OFF-SACs. The IPSCs were more noticeable in OFF- than in ON-SACs. Similar to reported retinal ganglion cell (RGC) oscillation in *rd1* mice, EPSC oscillation was synaptically driven by glutamate and sensitive to blockade of NaV channels and gap junctions. These data suggest that akin to *rd1* mice, AII-AC is a prominent oscillator in rhoΔCTA mice. Surprisingly, OFF-SAC but not ON-SAC EPSC oscillation could readily be enhanced by GABAergic blockade. More importantly, weakening the AII-AC gap junction network by activating retinal dopamine receptors abolished oscillations in ON-SACs but not in OFF-SACs. Furthermore, the latter persisted in the presence of flupirtine, an M-type potassium channel activator recently reported to dampen intrinsic AII-AC bursting. These data suggest the existence of a novel oscillation mechanism in mice with PD.

## Introduction

Pathological loss of photoreceptors is common in retinal degeneration and a leading cause of blindness. The resultant retinal dysfunction has been characterized in several rodent photoreceptor degeneration (PD) models at varying time points following photoreceptor loss. One consequence of PD is a spontaneous hyperactivity in inner retinal neurons, which has been observed in retinal ganglion cells (RGCs) of *rd1* mice (Stasheff, 2008; Menzler and Zeck, 2011; Poria and Dhingra, 2015), *rd10* mice (Goo et al., 2011; Biswas et al., 2014), and Royal College of Surgeons (RCS) rats (Sauvé et al., 2001). Heightened RGC spontaneous activity often manifests itself as a rhythmic oscillation in membrane potential that may lead to synchronous spiking patterns among RGCs of similar functional classes or to phase-shifted discharges among heterotypic RGC populations (Margolis et al., 2014). Although it is known that the RGC oscillations are driven synaptically (reviewed by Margolis and Detwiler, 2011), the precise mechanisms and origin of oscillation are not completely understood (Ye and Goo, 2007; Borowska et al., 2011; Trenholm et al., 2012; Yee et al., 2012; Choi et al., 2014). Nevertheless, it has been proposed that RGC oscillations in the *rd1* mouse model arise from intrinsically bursting AII-ACs (Choi et al., 2014) that transmit their synchronous rhythm to depolarizing ON cone bipolar cells (ONCBs) via a well-known gap junction coupling network (Borowska et al., 2011; Trenholm et al., 2012). The rhythmic activation of bipolar cells then produces rhythmic glutamatergic release at their axon terminals and ultimately the rhythmic activation of RGCs. This hypothesis is supported by the observation that inactivation of connexin 36 (Cx36), a key gap junction protein that plays a major role in the AII-AC gap junction network, eliminated the PD-induced hyperactivity *in vitro* in RGCs and *in vivo* in superior colliculus of *rd10* mice (Ivanova et al., 2015a). Moreover, oscillations mediated by the AII-AC network could also be measured in OFF RGCs either directly through activation of the OFF pathway or indirectly via OFF cone bipolar cells (OFFCBs) through sign-inverting glycinergic synapses (Poria and Dhingra, 2015).

The function of inner retinal hyperactivity in PD models remains unknown but it has been suggested that hyperactivity may help maintain RGC dendritic architecture following loss of photoreceptors (Mazzoni et al., 2008). Finding ways to retain retinal circuit integrity is desirable, but heightened activity likely reduces the signal-to-noise ratio in RGC outputs (Yee et al., 2012; Barrett et al., 2015). Current data on RGC oscillation in mice with PD, especially those from *rd1* mice, suggests that only a subset of RGCs exhibit rhythmic activity (Margolis et al., 2008; Stasheff, 2008; Borowska et al., 2011; Yee et al., 2014). The underlying reason remains unknown but it may result from different excitatory and inhibitory inputs to different RGC subtypes (Yee et al., 2012). Thus, dissecting retinal circuits driving various RGC subtypes with or without PD-induced oscillatory activity may provide unique insights into RGC diversity and assist in the development of novel therapeutic strategies.

Establishing the generality of RGC hyperactivity across PD models is imperative as it remains unclear whether heightened activity occurs in all PD models. Furthermore, it is unclear which remaining retinal neurons, including amacrine and bipolar cells, play a role in rhythm generation. A study by Jones et al. (2003) has systematically compared the progression of neuronal remodeling following photoreceptor loss in retinas of naturally occurring and genetically engineered animal models. Among the models examined, an autosomal dominant PD model originally named S334ter and hereon referred to as rhoΔCTA, which expresses a rhodopsin mutant with a premature truncation at Ser334 in retinal rods, was found to exhibit fast photoreceptor death and slow retinal remodeling, similar to those seen in *rd1* mice (reviewed by Jones and Marc, 2005). This autosomal dominant PD model has distinct advantages in studying the PD-induced inner retinal oscillation over other models because it is genetically more tractable and easier to combine with other genetic backgrounds. As RGC activity is controled by amacrine cells (ACs) through feedback and/or feed-forward inhibition (Masland, 2012), we focused our studies on these retinal interneurons. However, given that there are many AC subtypes in the retina, we began with the genetically tractable starburst amacrine cells (SACs), which are the sole source of acetylcholine in the retina capable of diffusely modulating RGC excitability (Schmidt et al., 1987) and play an essential role in retinal direction-selective circuits (reviewed by Wei and Feller, 2011). To do this we selectively expressed the fluorescent protein tdTomato in SACs in the rhoΔCTA background. This allowed us to target SACs for electrical recordings in whole mount retinas. Using this preparation, we found that heightened activity is robust and rhythmic in ON type SACs (ON-SACs) displaced in the ganglion cell layer (GCL). Pharmacological studies then showed that the interaction between AII-AC and ONCB generates oscillation in ON-SACs of rhoΔCTA retinas, as proposed for the *rd1* model. Unexpectedly, we also found that OFF-SACs whose somata reside in the inner nuclear layer (INL) oscillate but appear to be insensitive to pharmacological manipulations that aim to suppress AII-AC gap junction network or intrinsic bursting. Thus, our results strongly suggest the existence of an additional mechanism that contributes to inner retinal oscillation.

## Materials and Methods

### Animals

All experiments were conducted in accordance with the rules and regulations of the National Institutes of Health guidelines for research animals, as approved by the institutional animal care and use committee of Baylor College of Medicine. Acquired from the Jackson Laboratory, the Ai9 tdTomato reporter (B6.Cg-*Gt(ROSA)26Sor^tm9(CAG–tdTomato)Hze^*/J; Madisen et al., 2010) and the ChAT-IRES-Cre knock-in driver (B6;129S6-*Chat^tm2(cre)Lowl^*/J; Rossi et al., 2011) were bred into the rhoΔCTA background (Chen et al., 1995). The rhoΔCTA mouse was a different founder line generated by Jeannie Chen using the same transgenic construct that she used for the making of the published S334ter animals. It was previously used in a retina remodeling study (Jones et al., 2003) and is currently maintained in our labs in a mixed 129SvEv and C57BL/6J background. Genotyping was performed by PCR using primers suggested for the reporter and Cre driver as found on-line in the Jackson Laboratory website. The rhoΔCTA animals were genotyped using the following primers: RH2: 5′- TGG GAG ATG ACG ACG CCT AA and RH3: 5′- TGA GGG AGG GGT ACA GAT CC, with a 320 bp product indicating transgene presence. Additional genotyping confirmed the presence of wild type alleles at Pde6b (Pittler and Baehr, 1991) and GPR179 (Balmer et al., 2013) loci. All animals were kept on a 12/12 h light/dark cycle in microisolators with access to food and water *ad libitum*. For this study, 2–4 month-old male mice with complete degeneration of photoreceptors were used. Animals were deeply anesthetized with isoflurane inhalation, followed immediately by decerebration and harvest of retinas.

### Electrophysiology

Freshly isolated retinas were flat-mounted under room light with the ganglion cell side up and placed onto a punched nitrocellulose membrane. This allows visualization of retinal neurons through differential interference contrast and epifluorescence under an Olympus BX51WI fixed stage microscope (Olympus USA, Central Valley, PA, USA). The retina was superfused (3–3.5 ml/min) by carbogenated (95% O_2_ and 5% CO_2_) and temperature-controlled (34–35°C; Warner instruments TC-324B in-line heater) mammalian Ringer solution (in mM): 120 NaCl, 5 KCl, 25 NaHCO_3_, 0.8 Na_2_HPO_4_, 0.1 NaH_2_PO_4_, 2 CaCl_2_, 1 MgSO_4_ and 10 D-glucose. Neurons positive for tdTomato expression in both GCL and INL were targeted for recording using whole cell patch pipettes pulled from borosilicate glass (10–14 MΩ, King Precision Glass, Claremont, CA, USA). For whole cell current clamp recording, a potassium-based internal solution was used (in mM): 125 K-gluconate, 8 NaCl, 4 ATP-Mg, 0.5 Na-GTP, 5 EGTA, 10 HEPES and 0.2% biocytin (w/v), and pH was adjusted to 7.3 using KOH. Cesium-based internal solution containing (in mM): 100 Cs-methanesulfonate, 8 NaCl, 4 ATP-Mg, 0.5 Na-GTP, 5 EGTA, 10 HEPES and 0.2% biocytin (w/v), pH 7.3 adjusted with CsOH, was used for whole cell voltage clamp recording. The liquid junction potentials (20 mV between Ringer solution and the cesium-based solution, and 15 mV between Ringer solution and the potassium-based solution) were calculated using Clampex software (Molecular Devices) and corrected after current clamp recording and during voltage clamp recording. Recordings were performed using an AM2400 amplifier (A-M Systems, Sequim, WA, USA) driven by the WinWCP program developed and maintained by John Dempster (University of Strathclyde, Glasgow, UK). Signals were filtered at 5 kHz and sampled at 40 kHz. For current clamp recording, the cells were recorded at rest in free-run mode for 10–15 min without current injection. For voltage clamp recording, cells were held at −60 mV and repetitively clamped at the reversal potential of chloride (−75 mV) and the reversal potential of glutamate transmission (0 mV). Identities of recorded cells were confirmed *post hoc* by staining for biocytin and checking for the signature starburst morphology.

### Pharmacology

Drugs were bath-applied via perfusion. Picrotoxin (PTX, 50 μM) and (1,2,5,6-Tetrahydropyridin-4-yl)methylphosphinic acid (TPMPA, 10 μM) were used simultaneously to inhibit both of GABA_A_ and GABA_C_ receptors. Strychnine (1 μM) was used to block glycinergic transmission. Glutamatergic transmission was blocked by a cocktail of 6-Cyano-7-nitroquinoxaline-2,3-dione disodium (CNQX disodium, 10 μM) and D-(-)-2-Amino-5-phosphonopentanoic acid (AP5, 10 μM). Tetrodotoxin (TTX, 500 nM) was used to block voltage-gated sodium channels (NaV). Tubocurarine chloride (5 μM) was used to block nicotinic acetylcholine receptors. Meclofenamic acid (MFA, 100 μM) was used as a non-selective gap junction blocker. Dopamine hydrochloride (100 μM) was used to reduce gap junction coupling in AII-ACs. Flupirtine maleate (25 μM) was used to open the M-type potassium channels. All chemicals were purchased from Sigma-Aldrich or Tocris, prepared fresh and used within 24 h.

### Data Analysis

The power spectral density (PSD) between 0.5–55 Hz were calculated by fast fourier transform (FFT) of 10 randomly picked 5 s segments of recordings using OriginPro (Origin Lab, Northampton, MA, USA). Peak oscillation frequency in each PSD profile was verified by visual examination of the raw recording traces. The differences in peak frequency and corresponding power level produced by drug application were analyzed using the Pair-Sample *t*-test, and *p* <0.05 was considered to be significant and marked by asterisks in Figures 3–5.

## Results

**Figure 1:**
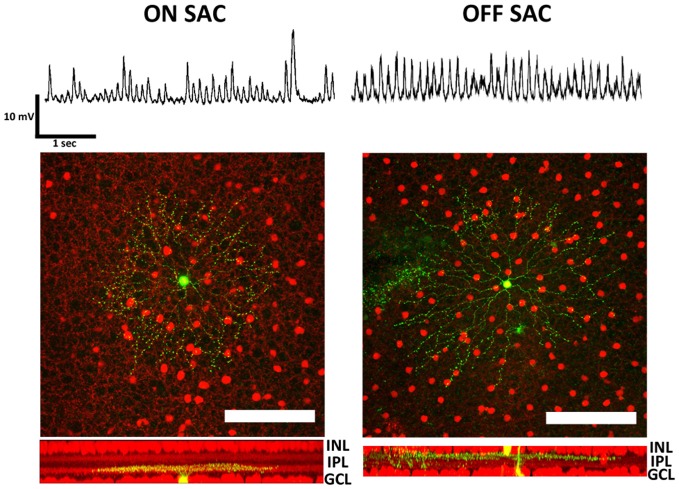
**Membrane potential oscillation in retinal ON- and OFF-SACs of the rhoΔCTA mouse**. Membrane potentials in both ON- and OFF-SACs showed robust membrane potential oscillation. Cells (green) targeted for patch clamp recordings in tdTomato expressing retinal cholinergic neurons (red). Cells in both GCL and INL showed typical SAC dendritic morphology and stratification levels in the IPL. GCL, ganglion cell layer; IPL, inner plexiform layer; INL, inner nuclear layer. Scale bar equals 100 μm.

To record from both SAC types, we introduced a ChAT-IRES-Cre driver and a tdTomato reporter into the rhoΔCTA background. Membrane potentials of both ON- and OFF-SACs were found to oscillate (Figure 1, top), similar to RGC oscillations previously described in *rd1* mice. We ensured that the neurons from which we recorded were SACs by performing *post hoc* morphological analysis. All the recorded neurons displayed signature SAC dendritic morphology and stratification levels in the inner plexiform layer (IPL; Figure 1, bottom).

**Figure 2:**
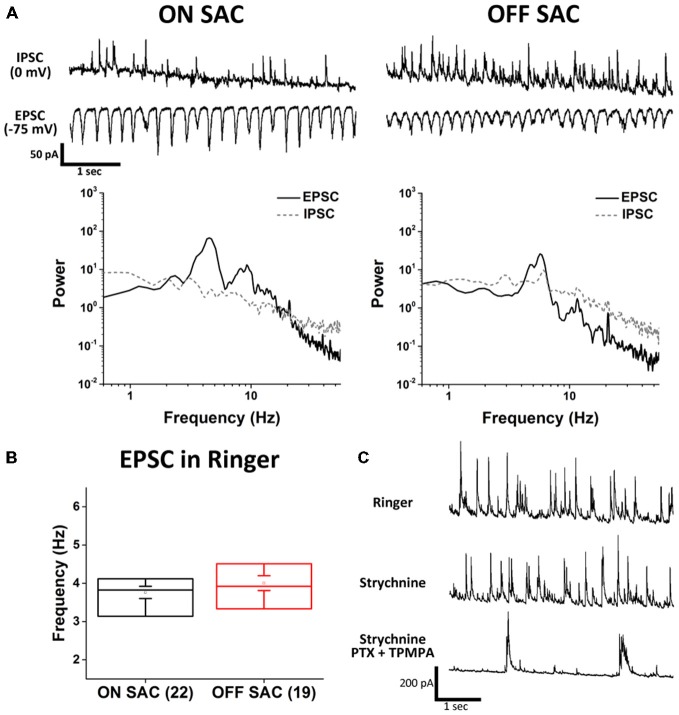
**Distinct excitatory and inhibitory inputs underlying membrane potential oscillation in ON- and OFF-SACs. (A)** Representative EPSCs and IPSCs from ON- and OFF-SACs with rhythmicity seen only in EPSCs and not in IPSCs. Frequency peaks of oscillation were evident in the power spectral density (PSD) following fast fourier transform (FFT) of recording data. **(B)** Averaged peak frequencies of EPSC oscillation are similar in ON- and OFF-SACs. Cell numbers are indicated in parentheses. Error bars = SEM. **(C)** Representative IPSCs seen in an OFF-SAC were inhibited by GABA antagonists but not by glycine receptor antagonist.

To investigate the mechanisms of SAC oscillation, we used a cesium-based intracellular solution and voltage clamp recordings to discern between EPSC and IPSC. To isolate EPSC, we clamped the membrane potential of SACs at the calculated reversal potential of chloride (−75 mV). To isolated IPSC, we voltage clamped SAC membrane potentials at the reversal potential for glutamate (0 mV). Using this strategy we found robust rhythmic EPSCs and arrhythmic IPSCs (Figure 2A). Although the proportion of EPSCs observed in ON- and OFF-SACs appeared similar, IPSCs were more frequently detected in OFF- SACs. We next quantified membrane current oscillations by performing an FFT analysis to determine the peak frequency of oscillation between 0.5–55 Hz. EPSCs from both ON- and OFF-SACs exhibited a peak frequency near 4 Hz (Figure 2B). There was no clear frequency peak in PSD of IPSCs. While EPSCs were driven by glutamatergic transmission (see below), IPSCs of the two types of SACs were driven predominantly by GABAergic transmission, as they were attenuated by a mixture of PTX (50 μM) and TPMPA (10 μM) but not by strychnine (1 μM; Figure 2C).

**Figure 3:**
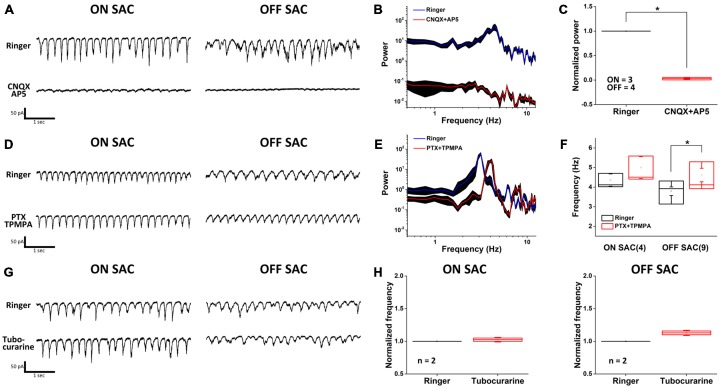
**Oscillation is driven by glutamatergic excitation and modulated by GABAergic inhibition. (A)** Rhythmic EPSCs in both ON- and OFF-SACs were attenuated by ionotropic glutamate receptor blockers. **(B)** A representative PSD profile of OFF-SAC EPSC oscillation. CNQX and AP5 (red) reduced power at all frequencies compared to those in normal Ringer solution (blue). The filled areas indicate SEM. **(C)** Reduction of power in both ON-and OFF-SAC EPSC oscillation peak frequency by ionotropic glutamate receptor blockers CNQX/AP5. Error bars = SEM. **(D)** Rhythmic EPSCs in both ON- and OFF-SACs persisted in the presence of GABAergic receptor antagonists. **(E)** A representative PSD of OFF-SAC EPSC oscillation in the presence of PTX and TPMPA (red) showing increased peak frequency compared to control (blue). The filled areas indicate the SEM. **(F)** Blockade of GABAergic transmission (middle panel) significantly enhanced the peak oscillation frequency in OFF-SACs but not in ON- SACs. **(G)** Removal of cholinergic synaptic inputs resulted in little/no change in the EPSC oscillation in both ON- and OFF-SACs. **(H)** Peak oscillation frequencies in both SACs types were not changed in the presence of tubocurarine. Cell numbers are indicated in parentheses. Error bars equal the SEM in **(A–F)** and SD in **(G–H)**.

To obtain a more comprehensive picture of how various neurotransmitters may contribute to SAC oscillation, we surveyed the effects of synaptic blockers on rhythmic EPSCs. We discovered that antagonists of ionotropic glutamate receptors (iGluRs; Figures 3A–C) significantly attenuated the oscillation in both SAC types. Furthermore, the polarity of EPSC oscillation in ON-SACs could readily be reversed when cells were held above 0 mV (data not shown), suggesting that iGluRs on ON-SACs are directly involved in oscillation. Thus it is likely that the periodic release of glutamate arises from upstream bipolar cells, including both depolarizing and hyperpolarizing types. Despite being the sole cholinergic neuron in the retina, EPSC oscillations in either SAC type were not affected by an exogenous cholinergic blocker (tubocurarine, 5 μM; Figures 3G,H). Furthermore, GABAergic inhibition impinging on SACs was not needed for oscillations (Figure 3D) but the frequency of OFF-SAC oscillation could be accelerated by GABAergic blockade (Figures 3E,F). We further noted that GABAergic blockade has no significant effect on ON-SAC oscillation.

**Figure 4:**
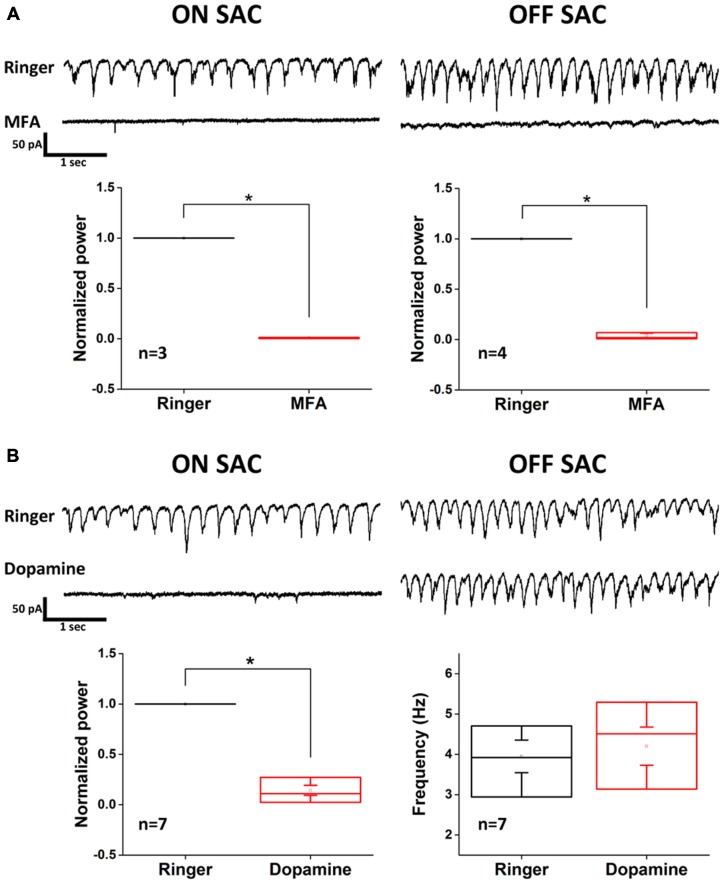
**Dopamine inhibits EPSC oscillation of ON- but not OFF-SACs. (A)** Oscillatory EPSCs in both ON- and OFF-SACs were eliminated after application of non-selective gap junction blocker MFA. Error bars indicate SEM. **(B)** Activation of dopaminergic receptors reduced the power of peak oscillation frequency in ON-SAC (bottom left panel). Peak frequency of OFF-SAC EPSC oscillation remained unchanged (bottom right panel). Error bars indicate the SEM.

The resemblance of SAC oscillation in the rhoΔCTA retina to that reported in RGCs of *rd1* and *rd10* retinas suggested that Cx36-mediated gap junction network among AII-ACs and between AII-ACs and ONCBs (Trenholm et al., 2012) might be involved in rhythmicity. To test this possibility, we used MFA (100 μM) to examine the dependance of SAC oscillation on gap junction coupling (Figure 4A). Rhythmic activity in both ON- and OFF-SACs was eliminated by MFA treatment, indicating that gap junction network plays an essential role in the rhoΔCTA model. However, MFA is a non-selective and non-specific gap junction blocker and there are many types of connexins in the retina. To seek further support for the involvement of AII-AC gap junction coupling in SAC oscillation, we turned to the reported modulation of Cx36 coupling in AII-ACs by dopamine. The activation of dopamine D1-like receptor reduced tracer coupling (Kothmann et al., 2009) by dephosphorylating the Ser293 residue of Cx36 in AII-ACs, while other gap junctions may remain permeable when dopamine is present (reviewed by Bloomfield and Völgyi, 2009). Figure 4B shows clearly that EPSC oscillation in ON-SACs (left panels) was disrupted in the presence of exogenous dopamine (100 μM), consistent with the speculation that the AII-AC network is involved. To our surprise, however, OFF-SACs remained oscillatory (Figure 4B, right panels), suggesting that another gap junction network insensitive to dopamine sustains OFF-SAC oscillation.

**Figure 5:**
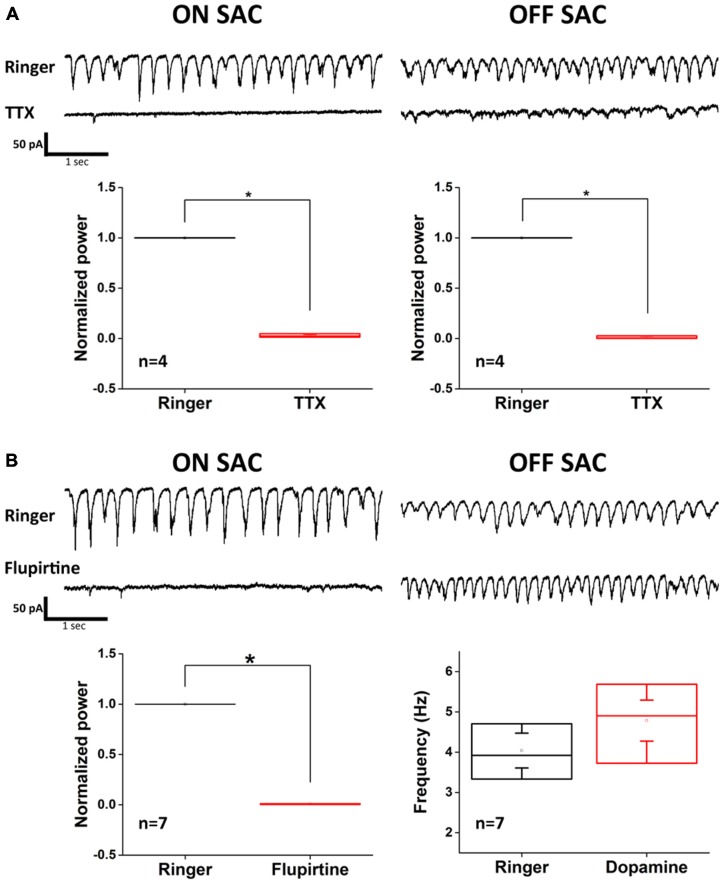
**Flupirtine inhibits ON-SAC but not OFF-SAC oscillation. (A)** Rhythmic EPSCs of both ON- and OFF-SACs were blocked in the presence of 500 nM TTX. Note that small, irregular EPSCs were remained in OFF-SACs but not in ON-SACs. Error bars indicate the SEM. **(B)** Inhibition of ON-SAC but not OFF-SAC oscillation by the M-type potassium channel opener flupirtine (25 μM). Significant reduction of power at peak oscillation frequency in ON-SACs was shown in the bottom left panel, while peak frequency of OFF-SAC oscillation remained similar to control (bottom right panel). Error bars indicate the SEM.

Because electrophysiological support for dopamine-mediated modulation of gap junction conductance between coupled AII-ACs is not found (Demb and Singer, 2012; Hartveit and Veruki, 2012), we further explored the different roles that the AII-AC gap junction network plays in the oscillation of ON- vs. OFF-SACs. To do this, we utilized a recently reported intrinsic bursting property of AII-ACs that can be blocked by TTX-mediated inhibition of NaV (Cembrowski et al., 2012) and by flupirtine-mediated activation of M-type potassium currents (Choi et al., 2014). Application of TTX (500 nM) effectively diminished EPSC oscillation in both SAC types (Figure 5A), although small and irregular activities were still observed in OFF-SACs in the presence of TTX. However, activating M-type potassium channels by flupirtine (25 μM) attenuated only EPSC oscillation in ON-SACs and not in OFF-SACs (Figure 5B). Taken together, the differential dopamine and flupirtine effects on the two SAC types strongly support a notion that OFF-SAC oscillation is through a mechanism different from the one driving RGC oscillation in *rd1* and ON-SACs in rhoΔCTA mice.

## Discussion

The rhoΔCTA mouse exhibits rapid loss of photoreceptors followed by slow onset of retinal remodeling (reviewed by Jones and Marc, 2005). We found oscillations of SAC membrane potential that require gap junction networks in the retina that are synaptically driven by glutamatergic transmission. The novelty of this investigation lies in the unexpected insensitivity of OFF-SAC oscillation to dopamine and flupirtine that distinguishes it from the mechanism involving the well-characterized AII-AC gap junction network that drives ON-SAC oscillation. Our findings indicate that not one but multiple mechanisms underlie inner retinal hyperactivity following PD. This work warrants a comprehensive survey of RGC diversity and may provide a means to catalog different RGC types according to their connections to distinct retinal circuits in PD animal models. Furthermore, identification of cellular components involved in the novel oscillation mechanism will be helpful in understanding the intricacy of retinal circuitry subserving normal vision.

### ON-SAC Oscillations in Wild Type and rhoΔCTA Mice

It was reported by Petit-Jacques et al. (2005) that a number of inner retinal neurons, including ON-SACs, displayed rhythmic inward currents in wild type retinas with a frequency similar to what we observed in ON-SACs of rhoΔCTA retinas. The rhythmic activity in wild type ON-SACs appeared to be driven similarly by synaptically released glutamate. Furthermore, the glutamate release depended on L-type voltage gated calcium channels and could be enhanced by blocking GABAergic transmission or by treating the retina with the non-selective voltage-gated potassium channel blocker Tetraethylammonium chloride (TEA; Petit-Jacques et al., 2005). The same group later reported another light-dependent and faster oscillatory current in wild type ON-SACs with properties similar to spontaneous oscillation (Petit-Jacques and Bloomfield, 2008). The light-dependent oscillation could be blocked by glutamate transporter antagonists or attenuated by GABAergic and glycinergic blockers and by TTX treatment. For the following reasons we consider the oscillation we observed in rhoΔCTA ON-SACs different from those of wild type retinas. First, wild type ON-SACs under our recording conditions did not have an obvious peak in PSD, suggesting that if and when they oscillate, it is not as robust as what is routinely seen in mice with PD (data not shown). However, these differences may be explained by the low concentration (1 mM) of TEA purportedly used in their original report to enhance the “small-amplitude” oscillations in approximately half of recorded SACs. Second, blockade of inhibitory transmission enhanced ON-SAC oscillation in wild type retinas but had no effect on oscillation of ON-SACs in rhoΔCTA mice. Third, TTX eliminated oscillation in our hands (Figure 5A), but only reduced oscillation amplitude or frequency in theirs (Figure 7 of Petit-Jacques and Bloomfield, 2008). Finally, spontaneous or light-enhanced wild type ON-SAC oscillation could be blocked by inhibiting L-type calcium channels by 30 μM Nifedipine, which has no effect on oscillation of ON-SACs in rhoΔCTA mice (Chen et. al, unpublished results).

While comparing ON-SAC oscillation between WT and rhoΔCTA mice may be a fruitful direction to examine the diverse synaptic connections underlying ON-SAC excitability, we shall focus the subsequent discussion solely on rhoΔCTA SAC oscillations, whose frequency is noticeably a bit lower than that reported in the *rd1* and *rd10* mouse RGCs and AII-ACs. An obvious question arises: is this due to a difference between SACs and other inner retinal neurons or a difference between rhoΔCTA and other mouse PD models? To answer this question, we have begun to examine oscillation of RGCs in rhoΔCTA retinas and found diversity of oscillation characteristics and distinctive sensitivities to pharmacological blockade in different RGC types (Chen et al, unpublished results). Yee et al. (2014) had done a similar but much larger survey of oscillating RGCs in *rd1* retinas and reported 10 RGC types that oscillated with diverse and distinctive characteristics. Therefore, it appears that SAC oscillation we observed in rhoΔCTA mice is not limited to just these two cell types and it is prevalent in many other inner retinal neurons.

### Inhibition of ON-SAC Oscillation in rhoΔCTA Mice by Dopamine and Flupirtine

The gap junction network of AII-ACs, either the homotypic type amongst themselves or the heterotypic type with ONCBs has been extensively studied. Exogenous dopamine suppresses Cx36 coupling by promoting dephosphorylation at its Ser293 residue (Kothmann et al., 2009). Our data shows that dopamine reversibly blocked ON-SAC oscillation. However, dopamine also triggers diverse actions through its receptors that are abundantly expressed in the retina and is known to modulate ion channel conductance (Pfeiffer-Linn and Lasater, 1996; Witkovsky, 2004; Ichinose and Lukasiewicz, 2007; Popova, 2014). Therefore, our finding that dopamine inhibited the ON-SAC oscillation may not prove the involvement of AII-AC network in SAC oscillation, especially in light of the noted lack of electrophysiological support for modulation of AII-AC gap junction coupling by dopamine (Demb and Singer, 2012; Hartveit and Veruki, 2012). Ivanova et al. (2015b) recently reported morphological and neurochemical aberrations in dopaminergic amacrine cells (DACs) in three mouse PD models and found reduced synaptic contacts between DACs and AII-ACs, raising a possibility that exogenous dopamine merely makes up for weakened modulation of AII-ACs by DACs. To investigate whether AII-ACs indeed play a role, we found that opening the slow and voltage dependent M-type potassium channels by low concentration (25 μM) of flupirtine reversibly blocked ON-SAC oscillations in the rhoΔCTA retinas. Activation of these potassium channels reportedly leads to AII-AC hyperpolarization to a point that its intrinsic bursting becomes inhibited, leading to suppression of RGC oscillation in *rd1* retinas (Choi et al., 2014). To further strengthen the notion that the AII-AC network is needed, we predicted and indeed found that low level of TTX (500 nM) robustly prevented ON-SACs from oscillating (Figure 5A). Finally, because MFA effectively blocked oscillation in our hands and a recent report showed the requirement of Cx36 for RGCs to oscillate in *rd10* mice (Ivanova et al., 2015a), we have obtained preliminary results from a few morphologically identified ON-SACs in three Cx36^−/−^/rhoΔCTA mutant mice that Cx36 is indeed required for ON-SACs in the rhoΔCTA background to oscillate (Chen et al., unpublished results). Taken together, these data leave little room to discount the requirement of AII-AC gap junction network in ON-SAC oscillation.

### Mechanism Underlying OFF-SAC Oscillation

The finding in Figures 4B, 5B that OFF-SAC oscillation was insensitive to pharmacological agents capable of blocking ON-SAC oscillation was an exciting observation. Being essential for direction-selective circuits and existing as mirror partners on both sides of the IPL, the mechanism underlying oscillation of OFF-SACs appeared to be different from that of ON-SACs. This finding joins a trending thought that retinal cholinergic neurons are not identical (Sun et al., 2013; Ishii and Kaneda, 2014; Borghuis and Fransen, 2015). Regarding the oscillation mechanism, because of its sensitivity to iGluR blockers, MFA, TTX, and more relevantly, insensitivity to strychnine, flupirtine and dopamine, it is reasonable to rule out the direct involvement of the AII-AC network in OFF-SAC oscillation. We speculate here that the periodic glutamate release at hyperpolarizing OFFCB terminals to be the oscillation source. The inhibitory effect of MFA suggests that gap junction coupling among presynaptic OFFCBs may be a possibility. It is known that several types of OFFCBs in mouse retina express connexins (Feigenspan et al., 2004; Hilgen et al., 2011) and at least one type of OFFCBs in rabbit retina has been shown to be gap junction coupled both homo- and heterologously (Mills, 1999). It is therefore of interest to know whether Cx36 is needed for OFF-SACs to oscillate as required in the AII-AC network. Hyperpolarization of AII-ACs resulting from gap junction blockade in *rd1* retina (Choi et al., 2014) may reduce the bursting of some types of OFFCBs with Ca^2+^-dependent regenerative activity (Ma and Pan, 2003). However, the insensitivity of OFF-SAC oscillation to flupirtine suggests that AII-AC hyperpolarization without gap junction uncoupling therein may not be enough to disrupt the OFFCB oscillatory activity. Furthermore, a recent report has identified a restricted set of bipolar cells that express D1 dopamine receptors (Farshi et al., 2015). Since exogenous dopamine has no effect on OFF-SAC oscillation, this suggests that type T1, T3b and T4 OFFCBs may play a negligible role here. We also suspect the involvement of other spiking ACs because of the inhibitory effect of TTX. It is equally possible that OFFCB oscillation is an intrinsic property of the cell, similar to that modeled for AII-ACs, which could be unmasked when outer retina activity is disrupted. While determining the exact oscillation mechanism and cellular components is important, it nonetheless falls beyond the scope of the present study. The notion that more than one oscillation generator exists in retinas following PD suggests that other PD models need to be tested to validate the generality of our findings. Nevertheless, our data indicate for the first time that oscillation of RGCs in PD models such as *rd1* and *rd10* mice are likely to be more complicated than previously reported and that current knowledge of inner retinal hyperactivity following PD may have seen just a small piece of a larger process.

### Oscillation as a Means to Probe Diversity of Inner Retinal Neurons

Many reports adhere to the idea that inner retinal neurons such as RGCs and ACs are diverse and each subserves vision in unique circuits (Masland, 2011). In published systematic surveys, from conservative to eager estimation, RGCs were clustered into 15–30 types (recently reviewed by Sanes and Masland, 2015) and ACs into around 40 types (MacNeil and Masland, 1998; MacNeil et al., 1999). As all surveys demand meticulous handling of large data sets and depend in varying degrees on morphological features and in some cases genetic markers (Huberman et al., 2009; Rivlin-Etzion et al., 2011; Trenholm et al., 2011), incorporating data on functional connection will conceivably add significantly to such efforts. As the diversity of RGCs in normal and diseased retina remains an active topic of vision research (Völgyi et al., 2009; Pang et al., 2015; Sanes and Masland, 2015), to efficiently harness RGC oscillation in mouse PD models as a classification tool is by all means an onerous task. It demands not only examination of a large number of retinal neurons but also the provision of means or reagents to allow reliable targeting of an identified RGC type with unique oscillation characteristics. Yee et al. (2014) has pioneered a study in this particular direction and defined 10 morphological RGC types in *rd1* retinas. With the knowledge that these oscillating neurons may possess differential sensitivity to dopamine or flupirtine, strategies for more in-depth investigation of inner retina oscillation may now be adopted to access neuronal diversity and circuit connectivity. The existence of more than one oscillation mechanisms operating at similar frequencies (Figure 1) also raises an intriguing possibility that an RGC may appear to be non-oscillating (Borowska et al., 2011) but in reality receives out-of-phase inputs from two sources that cancel each other out. Oscillation in this speculated condition can then be unmasked by pharmacological treatment. Using the recording conditions and mouse models depicted here, one may investigate similarity/dissimilarity of hyperactivity among different RGC types with relative ease. Combining the convenience of interrogating circuit connectivity described here with an ever-expanding collection of genetically marked neurons (Sanes and Masland, 2015) will inevitably have an impact on current and future census efforts for RGCs and ACs.

### Generality and Function of Neuronal Hyperactivity in Deafferentated Retinas

This investigation extends our general understanding of inner retinal hyperactivity to include a novel oscillation mechanism, an additional mouse PD model (rhoΔCTA) and to two more AC types (ON- and OFF-SACs). In a different study, we have gathered evidence that this may further be applied to hyperactivity in some mouse models lacking electroretinographic b-waves (Tu et al., 2014, 2015; Chen et al., 2015). One important question emerging from this generalization is whether inner retinal hyperactivity has any biological function, or merely a pathologic fallout following PD and a nuance to retinal prosthesis and gene/cell therapy fields. Experiments are currently underway to answer these questions.

## Author Contributions

Study design: H-YT and C-KC. Experimentation: H-YT, Y-JC, C-KC. Data analysis: H-YT, ARM, C-CC, C-KC. Manuscript preparation: H-YT, ARM, C-CC and C-KC.

## Funding

The work is supported by NIH grants EY013811 and EY022228 to C-KC, MH094626 and MH103695 to ARM, an NEI vision core grant EY002520 to the Department of Ophthalmology, Baylor College of Medicine, and an NSC grant NSC-101-2628-B-007-001-MY3 to C-CC.

## Conflict of Interest Statement

The authors declare that the research was conducted in the absence of any commercial or financial relationships that could be construed as a potential conflict of interest.
