# Corrigendum: A Novel Retinal Oscillation Mechanism in an Autosomal Dominant Photoreceptor Degeneration Mouse Model

**DOI:** 10.3389/fncel.2017.00257

**Published:** 2017-08-21

**Authors:** Hung-Ya Tu, Yu-Jiun Chen, Adam R. McQuiston, Chuan-Chin Chiao, Ching-Kang J. Chen

**Affiliations:** ^1^Department of Ophthalmology, Baylor College of Medicine Houston, TX, United States; ^2^Institute of Molecular Medicine, National Tsing Hua University Hsinchu, Taiwan; ^3^Department of Life Science, National Tsing Hua University Hsinchu, Taiwan; ^4^Department of Anatomy and Neurobiology, Virginia Commonwealth University Richmond, VA, United States; ^5^Institute of Systems Neuroscience, National Tsing Hua University Hsinchu, Taiwan; ^6^Department of Biochemistry and Molecular Biology, Baylor College of Medicine Houston, TX, United States; ^7^Department of Neuroscience, Baylor College of Medicine Houston, TX, United States

**Keywords:** starburst amacrine cell, retina, photoreceptor degeneration, AII amacrine cell, oscillation mechanism

There was a mistake in the x-axis label in Figure 5B as published. The corrected Figure 5 appears below. The authors apologize for the typographical mistake. This error does not change the scientific conclusions of the article in any way.

**Figure 5:**
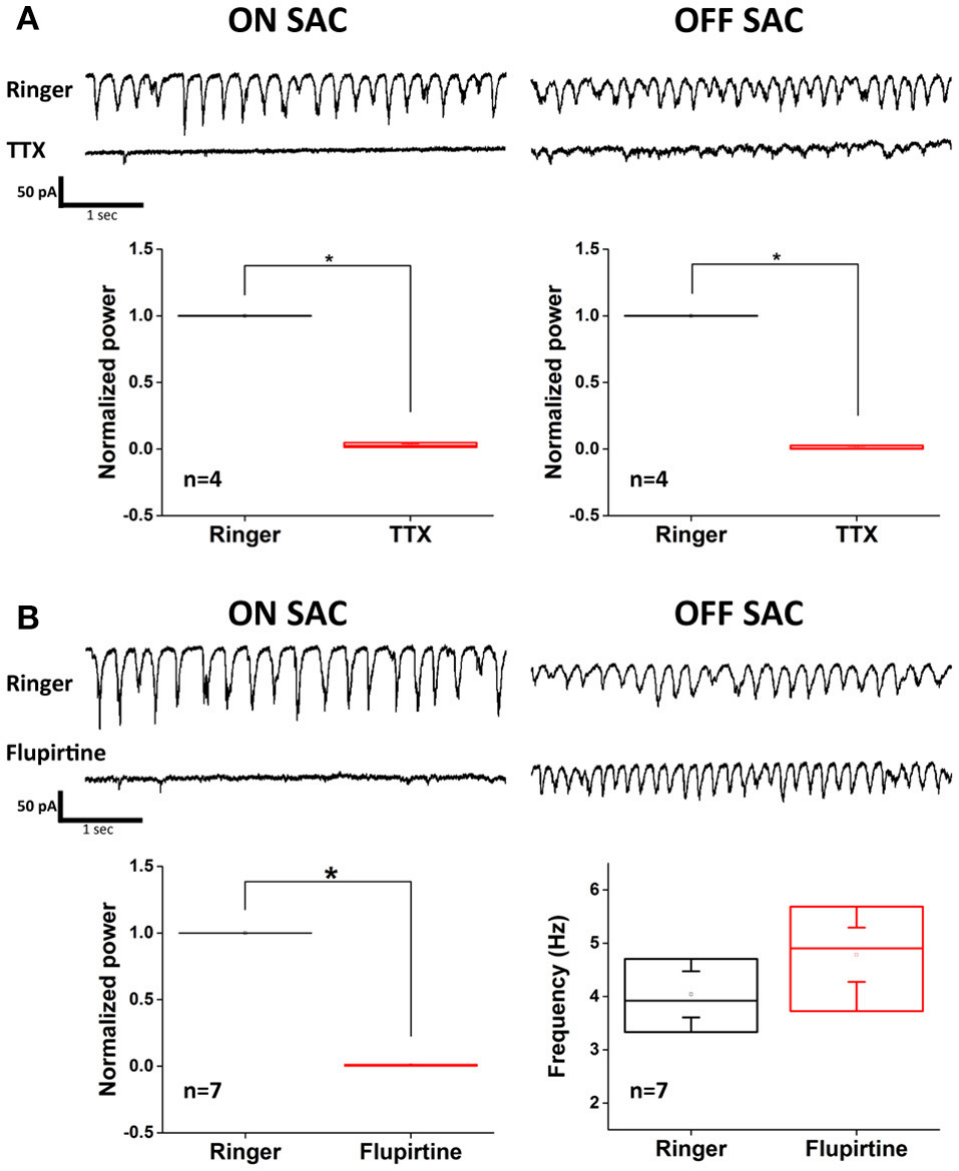


## Conflict of interest statement

The authors declare that the research was conducted in the absence of any commercial or financial relationships that could be construed as a potential conflict of interest.

